# The Mediating Effect of Disability Acceptance in Individuals with Spinal Cord Injury Participating in Sport for All

**DOI:** 10.3390/ijerph182010883

**Published:** 2021-10-16

**Authors:** Hyoyeon Ahn, Keunchul Lee, Youngho So

**Affiliations:** 1 Department of Physical Education, College of Education, Seoul National University, Seoul 08826, Korea; ahy0522@snu.ac.kr; 2 Department of Physical Education, College of Natural Science, Changwon National University, Changwon 51140, Korea; 3 Department of Kinesiologic Medical Science, Dankook University, Cheonan 31116, Korea

**Keywords:** spinal cord injury, disability acceptance, belongingness, self-efficacy, life satisfaction

## Abstract

The purpose of this study is to examine the importance of disability acceptance among individuals with spinal cord injury (SCI) participating in the Sport for All program through self-help group activities with other individuals with SCI. This study investigated whether disability acceptance mediates the relationship between self-efficacy and life satisfaction and between sense of belonging and life satisfaction. Subjects were 142 individuals with SCI participating in the self-help group with other sports activities including para table tennis, swimming, wheelchair rugby, and weight training. A simple mediation effect analysis showed that disability acceptance significantly mediated the relationship between self-efficacy and life satisfaction (indirect effect, *b* = 0.219) and between the sense of belonging and life satisfaction (indirect effect, *b* = 0.289). The results suggest the importance of disability acceptance for individuals with SCI participating in “Sports for All” programs.

## 1. Introduction

Most spinal cord injuries (SCI) result in paralysis in the body from damage to the spinal cord due to an unexpected accident. Approximately 80% of individuals with SCI depend on a wheelchair [1]. SCI may lead to a reduction or cessation in participation in physical activities and is associated with an increased risk of secondary physical problems, such as obesity and bedsores [2,3,4,5].

According to the standards disclosed by the Korea Ministry of Health and Welfare [6] and the Korea Institute for Health and Social Affairs [7], the population of individuals with SCI in Korea was estimated to be 54,000. The Korean Ministry of Health and Welfare reported that for 90.5% of the population with severe disabilities, such as those with SCI, most injuries were due to traffic or industrial accidents [8]. Similarly, according to the report of the National Center for Spinal Cord Injury Statistics, 91.9% were reported to be caused by traffic accidents, falls, or violence [9]. Such acquired disabilities can, at times, be accompanied by psychological problems, such as anxiety, because of the sudden and permanent loss of physical functions [10], as well as depression, anger, hostility, and violent actions [11]. For example, individuals with SCI may experience recurrent trauma from the accident, which has been shown to be associated with major depressive disorder [12]. Thus, it is considered important to approach these psychological factors for individuals with SCI.

Recently, research into the effects of sports and rehabilitation program participation among individuals with SCI have expanded from treating mainly the physical disabilities to also considering psychological recovery and improving patients’ quality of life [13]. Most of the early studies on individuals with SCI focus on the effects of counseling or rehabilitation programs through qualitative research methods, application of tele-counselling [14], exploring the quality of life [15,16], and neuropathic pain [17]. Although considerable research attention has highlighted the effects of participating in sports activities of individuals with SCI, several authors have noted the lack of quantitative research addressing individuals with SCI [18,19]. Moreover, most individuals with SCI have an acquired disability and may have an especially difficult time in accepting their disability compared to those born with a disability [20]. Therefore, the aspect of psychological approach and disability acceptance are important in research on individuals with SCI.

The literature on the psychological recovery of individuals with disabilities typically emphasizes the goal of increasing life quality or satisfaction through social integration [21,22]. Roth, Zittel, Pyfer, and Auxter [23] revealed that that social integration, through sports or physical activities, is essential to increase the self-esteem of individuals with disabilities. In addition, participation in sports activities may help them overcome pain or sadness [24] and it can positively influence acceptance of one’s disability [25]. Disability acceptance is largely subjective [26,27] and is associated with positive psychological outcomes, such as life satisfaction [28,29], self-esteem [30], and self-conception [31]. Reflecting previous studies, in participation through a self-help group, where participants help each other in similar situations, perhaps having a disability becomes less critical [32,33]. Lee and Stephen [34] reported that the social support and disability acceptance of individuals with SCI were improved through participation in sports activities.

For individuals with SCI, participation in sports and physical activities is associated with various positive psychological outcomes, such as social support [34], self-efficacy and psychological well-being [35,36,37]. Therefore, in this study, we try to approach the situation and perspective of individuals with SCI through self-efficacy that has already been revealed through previous studies. For example, in the SEQRS (Self-efficacy for Quad Rugby Skills) questionnaire [38], the self-efficacy construct was modified to fit diverse disabilities in the development process and situations related to sports or physical activities. Notably, it is important to consider the environmental factors, as in the current study.

Other factors that can affect quality of life and life satisfaction in individuals with severe physical disabilities are environmental factors such as social support [39,40]. In previous studies, peer- or self-help groups have helped to provide emotional support between the different types of received social support [41] and have enhanced individuals’ self-efficacy beliefs [42]. The types of received social support (physical, emotional, and informational support) can also diminish the negative effects of stress and mend psychological health [43]. In fact, the role of self-help group activity is important for individuals with SCI who need help when getting into a swimming pool or when using weight-training devices. Like Villie et al. [44], we operationalized social support as a “sense of belonging.” Social/psychological effects from such self-help group activities can give participants a “sense of belonging”, which can be defined as a perceived oneness with an organization and the experience of the organization’s successes and failures as one’s own [45]. Rude and Burham [46] explained that individuals who lack a sense of belonging can possibly become depressed because of pathological social behaviors such, as excessively dependence on others. Thus, one can predict that a sense of belonging can develop through daily sports and self-help group activities, which can have a positive psychological effect on individuals with SCI and contribute to the goal of psychological recovery.

**Figure 1:**
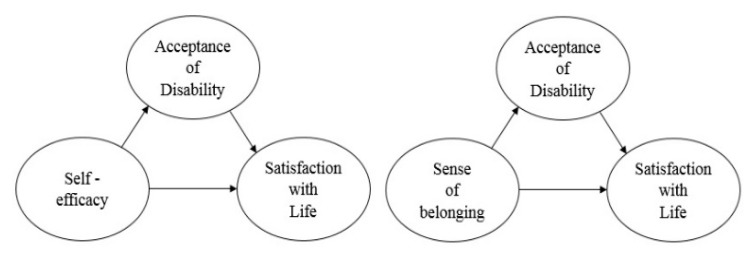
Conceptual model of study.

Therefore, in this study, we examined the importance of disability acceptance among individuals with SCI participating in Sport for All programs through self-help activities with other individuals with SCI. We also examined the positive role of disability acceptance in the relationship between self-efficacy, sense of belonging, and life satisfaction among individuals with SCI participating in Sports for All. This approach will help clarify the psychological effects of sports participation among individuals with SCI rather than focusing on physical activity programs. The conceptual model of our study is shown in Figure 1. Our specific research questions were as follows:

(1) What affect does disability acceptance have on the relationship between self-efficacy and life satisfaction?

(2) What affect does disability acceptance have on the relationship between sense of belonging and life satisfaction.

## 2. Methods

### 2.1. Participants

In this study, research participants participating in self-help group sports activities were recruited through convenience sampling. The participants were 142 individuals (110 males and 32 females, mean age = 41.13 ± 9.25 years) with SCI living in one of five cities in South Korea who regularly participated at least once a week in Sports for All programs, which is a self-help group provided by the Korea SCI Association, Gomduri Sports Center (a Korean national support rehabilitation facility), and the Korea Wheelchair Rugby Association. Participants were selected based on their regular participation (i.e., more than once a week) in Sports for All programs for over a year (mean times per month = 9.44 ± 6.29; mean duration = 4.73 ± 3.24 years). There were 15 sports event programs, such as wheelchair basketball, wheelchair rugby, and wheelchair table tennis. Every Sports for All programs comprises individuals with SCI, and physical activities are conducted in a way that participants are encouraged to help each other.

### 2.2. Measurement Items and Methods

#### 2.2.1. Self-Efficacy

Cha’s Korean General Self-efficacy scale [47], based on Bandura’s self-efficacy theory [48], was used to measure general self-efficacy. It comprises three factors (self-regulation, self-confidence, and task difficulty) and 24 items, which are measured using a 6-point Likert-style scale. However, since the other two questionnaires (i.e., in this study) consisted of 5-point Likert scales, we revised the Korean General Self-efficacy scale to a 5-point Likert scale (strongly disagree, disagree, neutral, agree, and strongly agree) to minimize the confusion among participants. The scale has been reliability and validity in previous studies (Cronbach’s alpha: 0.79–0.86; factor loading: 0.48–0.68) [49,50].

#### 2.2.2. Disability Acceptance

The Acceptance Disability Scale-Revised (ADS-R) was used to measure disability acceptance in individuals with SCI. The scale was created by Linkowski [51] then modified by Linkowski and Groomes [52]. The Linkowski’s [51] original scale comprised a single factor and 50 items; however, in Linkowski and Groomes [52], there were four factors and 32 items that were responded to on a 4-point Likert scale (strongly disagree, disagree, agree, and strongly agree) as opposed to the 6-point Likert format in the original scale. The four factors included an enlargement of scope of value, subordination of physique, containment of disability effects, and transformation from comparative value to asset value. We used Kim and Jo’s [53] translated questionnaire and the scale has a verified reliability and validity in their study (Cronbach’s alpha: 0.74, multicollinearity: 0.137~0.392 < |0.04|).

#### 2.2.3. Sense of Belonging

To measure the sense of belonging, we used the concept of “organizational identification,” which was defined by Mael and Ashforth [45], as an individual’s awareness as a member of an organization that they identify as being a part of. Only the items related to a sense of belonging were extracted and utilized in Kim’s [54] organizational identification questionnaire and translated into Korean. The scale consists of six items that are responded to on a 5-point Likert scale (strongly disagree, disagree, neutral, agree, and strongly agree). This questionnaire consists of questions about the success or criticism of the group.

#### 2.2.4. Life Satisfaction

The Satisfaction with Life Scale (SWLS) was used to access life satisfaction. The scale was created by Diener et al. [55], then modified by Kwon [56]. It comprises five questions that are responded to using a 5-point Likert scale (strongly disagree, disagree, neutral, agree, and strongly agree). We adapted the items to be appropriate for individuals with SCI (delete an item, “If I could my life over, I would change almost nothing”).

### 2.3. Procedure

Before study commencement, a sports psychology expert and adapted physical education expert reviewed the questionnaire for content validity (one is a professor of sports psychology, and the other is a Ph.D. researcher at the National Rehabilitation Center in South Korea). Researchers completed a research ethics education course at the Collaborative Institutional Training Initiative (CITI). This study was approved by the institutional review board (IRB) of Changwon National University, Korea (approval number: 7001066-202008-HR-020). Next, with the cooperation of Gomduri Sports Center, a Korean national support rehabilitation facility, and Sports for All self-help groups operated by the Korea SCI Association and Korea Wheelchair Rugby Association, the online and offline surveys were conducted to recruit participants across five cities. Potential participants were informed of the study purpose and were asked to provide their consent to participate. The questionnaire took approximately 25–30 min to complete (including time to explain the survey and study purpose). When participants could not write their responses, assistants provided help.

### 2.4. Data Analysis

Of the 169 questionnaires received, 27 were excluded due to incomplete or unclear responses (including multiple responses). Thus, data from 142 individuals were used for the analyses. The SPSS (version 18.0, IBM, New York, NY, USA) and PROCESS macro for SPSS [57] were used to conduct statistical analyses. First, a test of the measurement model was performed to investigate the stability of the research model and to check the scales’ construct validity of the scale. Next, descriptive statistics were analyzed to understand the overall characteristics and the normality of the data. Internal consistency reliability was calculated for the measurement scales using Cronbach’s alpha. Finally, to test the relationships between the variables, first Pearson’s correlations were analyzed, and then simple mediation effect tests were conducted to identify the mediating effect of disability acceptance. The mediating effect analyses were conducted using ordinary least squares (OLS). The OLS is a method of estimating the regression constant and regression coefficient by minimizing the residual sum of squares. In other words, the regression constant and coefficient are estimated to determine the best fit model of the data by using least squares criteria. The statistical significance level was set at 0.05.

## 3. Result

### 3.1. Measurement Model Testing

The reliability and validity of the measurement model consisted of four latent variables (self-efficacy, sense of belonging, disability acceptance, and life satisfaction) and eighteen observed variables (self-regulation, self-confidence, task difficulty preference, subordination of physique, containment of disability effect, transformation from comparative values to asset values, enlargement of scope of value, sense of belonging (6 items), and life satisfaction (5 items)). A confirmatory factor analysis with maximum-likelihood estimation indicated that the measurement model’s fit to the data was not satisfactory (χ^2^ = 410.470, df = 129, *p* < 0.001, Tucker-Lewis index(TLI) = 0.851, comparative fit index(CFI) = 0.874, root mean square error of approximation(RMSEA) = 0.124). In general, the RMSEA index is a good fit when it is below 0.08 [58], and the TLI and CFI indices are good at 0.90 or better [59,60].

Therefore, variables with a low factor loading values were not grouped as latent variables and retained as observed variables. In this process, three items were deleted: one for sense of belonging (item 2), one for life satisfaction (item 5), and an “enlarging the scope of values” variable for disability acceptance. Concurrently, a modification index was used to assume one correlation within the same latent variable (sense of belonging items 4 and 5). The results of this analysis indicated that the model had a satisfactory fit to the data (χ^2^ = 169.199, df = 83, *p* < 0.001, TLI = 0.939, CFI = 0.951, RMSEA = 0.086). The reason the RMSEA value is slightly higher in this result is because the sample number was relatively small.

The construct reliability (CR), average variance extracted (AVE) of each latent variable, and correlation between each concept are shown in Table 1. The CR of each latent variable was calculated using the formula suggested by Fornell and Larcker [61] and ranged from 0.873 to 0.963, exceeding the cutoff value (≥0.70). The AVE was calculated using the formula proposed by Hair, Black, Babin, Anderson, and Tatham [62] and ranged from 0.660 to 0.816, exceeding the cutoff value (≥0.50) in all cases. In addition, the AVE values were greater than the root values (*ϕ*) of the correlation coefficients between variables. Overall, there was no problem with the convergent and discriminant validity of the measurement model. Therefore, the validity of the proposed research model was obtained.

### 3.2. Descriptive Statistics, Reliability, and Correlation Tests

To check the basic information for each observed variable, descriptive statistics were examined. All results were less than the criterion values [63]: means (≤4.5), standard deviations (≤2.0), skewness (≤2.0), and kurtosis (≤4.0). Therefore, the assumption of normality was satisfied for all variables. The internal consistency (Cronbach’s α) for the instruments used in this study were relatively high (0.829–0.909; see the diagonal in Table 2). Correlation coefficients for the relationships between each sub-variable are presented in Table 2. Positive relationships are evident among all the sub-factors (*r* = 0.179–0.865).

### 3.3. The Mediating Effect of Disability Acceptance: Self-Efficacy and Life Satisfaction

Through the OLS analysis, Table 3 shows the results of the predictor variable for the mediator and dependent variable in the mediation model. Self-efficacy was positively related to disability acceptance (*b* = 0.609, *p* < 0.001) and life satisfaction (*b* = 0.658, *p* < 0.001). Furthermore, disability acceptance was positively associated with life satisfaction (*b* = 0.466, *p* < 0.001). Finally, Table 4 presents the results of the indirect effect analysis. The confidence intervals for the indirect effect (*b* = 0.289) of self-efficacy on life satisfaction through disability acceptance did not include zero (95% confidence interval (CI) = 0.0934–4.603); thus, the mediating effect was significant.

### 3.4. The Mediating Effect of Disability Acceptance: Sense of Belonging and Life Satisfaction

Table 5 shows the results of the relationship between sense of belonging, disability acceptance, and life satisfaction. Disability acceptance was positively predicted by sense of belonging (*b* = 0.237, *p* < 0.001). Sense of belonging and disability acceptance was positively related to life satisfaction (*b* = 0.385 and 0.925, respectively; *p* < 0.001). Lastly, Table 6 shows the results of the indirect effect analysis. The confidence intervals for the indirect effect (*b* = 0.219) of sense of belonging on life satisfaction through disability acceptance did not include zero (95% CI = 0.0934–4.603); thus, the mediating effect was significant.

## 4. Discussion

Lots of research indicates there are psychological benefits to participation in physical rehabilitation and sports [14,34,35]. Although most studies recognize the psychological benefits of physical activities and participation in sports for individuals with SCI, there is only a limited explanation concerning how psychological recovery can occur because of extraneous, mediating, and moderating variables. Thus, based on the results and limitations of preceding studies, we revealed how participation in sports-related self-help groups can aid in the psychological recovery of individuals with SCI. Specifically, we determined that self-efficacy and sense of belonging were positively related to life satisfaction in individuals with SCI participating in Sports for All programs. In addition, we clarified the role of the disability acceptance for individuals with SCI.

Our findings confirmed the mediating effects of disability acceptance in participation self-help group sports activities. Specifically, as in previous studies, the relationships between self-efficacy and life satisfaction, self-efficacy and disability acceptance, belonging and life satisfaction were confirmed. Moreover, we found that higher life satisfaction could be obtained through the mediating variable of disability acceptance for individuals with SCI. The need for efforts to resolve the complexity of these secondary conditions and their inter-relationships has already been suggested [13], and the mediating effects of disability acceptance explains the key relationships determined in this study. These results support Nicholls et al.’s argument that disability acceptance is a key factor in the recovery of people with spinal cord disorders [64].

Previous studies related to disability acceptance revealed that perceived disability is the strongest predictor of one’s adaptation and that perception has a greater impact on socio-psychological adjustment than actual impairment does [65,66]. Especially, Wright [67] explained that those with acquired disabilities, such as SCI, experience a sense of loss, and our study supports this notion. The is because most individuals with SCI have many wheelchair-related barriers and obstacles, which can limit their participation in physical activity [36]. In fact, oftentimes, they require third-party assistance to participate in Sports for All programs, such as assistance with getting into a swimming pool or using weight training device.

These results support the necessity of considering the psychological aspect of disability acceptance along with the physical activities involved in the rehabilitation processes of individuals with SCI. Our results also suggest that participation in self-help groups and group activity programs can increase disability acceptance for individuals with SCI, which can have vast psychological benefits. Through the self-help group sports activities, the acceptance of physical conditions and blocking the negative effects of disabilities can be increased, and ultimately, it will be possible to have a more positive effect on life satisfaction. Such findings have implications for self-help groups and those designing Sports for All programs. In other words, participating in self-help group sports activities that can help and support other individuals with SCI has a positive effect on disability acceptance; receiving social support [40] can diminish the negative effects of stress and mend psychological health [43]. This approach is the same as the concept of disability acceptance proposed by Linkowski [51,52]. Through the result of this study, it is possible to emphasize the need to increase specialized sport programs and facilities for individuals with a disability.

Although the results of this study are an important initial step to understanding the disability acceptance of individuals with SCI through self-help group participation in Korea, the results should be interpreted considering several limitations. First, this study was conducted without a comparison group and cross-sectional design. Thus, there are limitations in generalizing how sports and physical activity participation may affect the psychological variables collected in this study to a broader population. Although this study found the mediating effect of disability acceptance, it is necessary for future studies to verify whether sports activities are related to the psychological state by a comparative analysis, such as type of group—leisure and rehabilitation, self-help group or not. Moreover, Longitudinal research that observes the changing psychological variables during actual physical activity or sports is needed. Second, in this study, we did not consider the level of participation in Sports for All programs (i.e., time per month, duration, quality of the participation) and conducted research on participants within the Sports for All program. Thus, to allow for results generalization, the moderated mediating model should be verified with variables such as the level of sports participation or engagement. It is necessary to develop an approach that fosters positive psychological states in individuals with SCI through physical activity or sports participation. Future studies might, thereafter, effectively focus not only on finding methods of enhancing individuals, but also on modifying participants’ environments, such as the type of group and disability.

## 5. Conclusions

This study revealed the role of disability acceptance in individuals with SCI participating in Sports for All programs that comprised self-help group activities. Specifically, self-efficacy and sense of belonging were positively related to life satisfaction, which was enhanced by disability acceptance. The results provide opportunities to improve psychological rehabilitation efforts through participation in sports and physical activity with people in similar situations. Consequently, individuals with SCI can increase their life satisfaction through psychological rehabilitation and may acquire a more positive evaluation of their physical and psychological problems. As such, when designing sport programs for individuals with SCI, the inclusion of self-help group activities and environments promoting disability acceptance should be considered, rather than only focusing on participation in physical activity programs.

## Figures and Tables

**Table 1:** Construct reliability and AVE of measurement model Construct reliability.

Concept	Construct Reliability	AVE	Correlation between Concept (*ϕ*)			
			1	2	3	4
1. Self-efficacy	0.873	0.681	1	—	—	—
2. Sense of belonging	0.907	0.660	0.447 (0.199)	1	—	—
3. Acceptance of Disability	0.963	0.816	0.758 (0.574)	0.315 (0.099)	1	—
4. Satisfaction with Life	0.876	0.713	0.734 (0.539)	0.504 (0.254)	0.681 (0.464)	1

(*ϕ*): Root value of correlation coefficients between concepts, AVE = Average Variance Extracted.

**Table 2:** Description statistics and correlations among the variables.

Variable		M(SD)	Skewness	Kurtosis	1	2	3	4	5	6	7	8
Self-efficacy	SRE	3.36(0.97)	0.024	−0.792	0.900	—	—	—	—	—	—	—
	SC	3.44(0.75)	0.062	−0.454	0.735 **	0.831	—	—	—	—	—	—
	TDP	2.87(1.04)	0.272	−0.643	0.604 **	0.684 **	0.876	—	—	—	—	—
Sense of Belonging		4.10(0.87)	−0.822	0.030	0.412 **	0.404 **	0.371 **	0.909	—	x	—	—
Acceptance of disability	SP	2.69(0.75)	0.133	−0.733	0.516 **	0.666 **	0.498 **	0.179 *	0.829	—	—	—
	CDE	2.77(0.69)	0.161	−0.597	0.645 **	0.744 **	0.587 **	0.323 **	0.811 **	0.890	—	—
	TCVAV	2.89(0.65)	−0.046	−0.497	0.670 **	0.795 **	0.636 **	0.400 **	0.773 **	0.865 **	0.869	—
Satisfaction with life		3.33(1.03)	−0.110	−0.768	0.745 **	0.636 **	0.568 **	0.504 **	0.530 **	0.701 **	0.695 **	0.905

Alpha values on diagonal, correlation values below diagonal, * *p* < 0.05, ** *p* < 0.01, SD = standard deviation. SRE = self-regulation, SC = self-confidence, TDP = task difficulty preference, SP = subordination of physique, CDE = containment of disability effect, TCVAV = transformation from comparative values to asset values.

**Table 3:** Mediation effect of acceptance of disability between self-efficacy and satisfaction with life.

Predictor	*b*	SE	*t*	LLCI (95%)	ULCI (95%)
Outcome = Acceptance of disability (*R*^2^ = 0.574, *p* < 0.001)					
Self-efficacy	0.609	0.147	13.73 ***	0.5210	0.6962
Constant	0.820	0.044	5.56 ***	0.5285	1.1113
Outcome = Satisfaction with life (*R*^2^ = 0.576, *p* < 0.001)					
Self-efficacy	0.658	0.109	6.06 ***	0.4430	0.8721
Acceptance of disability	0.466	0.135	3.45 ***	0.1993	0.7334
Constant	−0.085	0.260	−0.33	-0.5999	0.4295

*** *p* < 0.001. *b* is an unstandardized parameter with SE. SE = Self-efficacy. LLCI = Lower level confidence interval, ULCI = Upper lever confidence interval.

**Table 4:** Index of indirect effect.

Indirect Effect	*b*	Boot SE	LLCI	ULCI
SE → AD → SL	0.289	0.0938	0.0934	0.4603

Bootstrap Sample = 10,000/LLCI = Lower level confidence interval, ULCI = Upper lever confidence interval. *b* is an unstandardized parameter with SE. SE = Self-efficacy, AD = Acceptance of disability, SL = Satisfaction with life.

**Table 5:** Mediation effect of acceptance of disability between sense of belonging and satisfaction with life.

Predictor	*b*	SE	*t*	LLCI (95%)	ULCI (95%)
Outcome = Acceptance of disability (*R*^2^ = 0.0992, *p* < 0.001)					
Self-efficacy	0.237	0.060	3.93 ***	0.1175	0.3558
Constant	1.81	0.253	7.17 ***	1.3119	0.3558
Outcome = Satisfaction with life (*R*^2^ = 0.556, *p* < 0.001)					
Self-efficacy	0.385	0.071	5.39 ***	0.2437	0.5259
Acceptance of disability	0.925	0.095	9.74 ***	0.7373	1.113
Constant	−0.821	0.332	−2.47	−1.4775	−0.1636

*** *p* < 0.001. *b* is an unstandardized parameter with SE. LLCI = Lower level confidence interval, ULCI = Upper lever confidence interval.

**Table 6:** Index of indirect effect.

Indirect Effect	*b*	Boot SE	LLCI	ULCI
SB → AD → SL	0.219	0.0664	0.1015	0.3657

Bootstrap Sample = 10,000/LLCI = Lower level confidence interval, ULCI = Upper lever confidence interval. *b* is an unstandardized parameter with SE. SB = Sense of belonging, AD = Acceptance of disability, SL = Satisfaction with life.

## Data Availability

The data are available upon request from the corresponding author.

## References

[1] Post M.W.M., Van Asbeck F.W.A., Van Dijk A.J., Schrijvers A.J.P. (1997). Services for spinal cord injured: Availability and satisfaction. Spinal Cord.

[2] Gélis A., Dupeyron A., Legros P., Benaim C., Pelissie J., Fattal C. (2009). Pressure ulcer risk factors in persons with spinal cord injury Part 2: The chronic stage. Spinal Cord.

[3] Jin Y.S., Han G.S., Choi S.K., Kim U.S. (1993). The effects of physical activity on serum lipid in paraplegia. Korean J. Sports Med..

[4] Nash M.S. (2005). Exercise as a health-promoting activity following spinal cord injury. J. Neurol. Phys. Ther..

[5] Tasiemski T., Kennedy P., Gardner B.P. (2006). Examining the continuity of recreation engagement in individuals with spinal cord injuries. Ther. Recreat. J..

[6] Kim S.H., Lee Y.H., Oh W.C., Hwang J.H., Oh M.A., Lee M.K., Lee N.H., Oh D.E., Kang D.W., Kwon S.J. (2017). The National Survey of the Disabled Persons.

[7] Korea Ministry of Health and Welfare (2019). The Ministry of Health and Welfare White Book.

[8] Kim S.H., Byun Y.C., Son C.K., Lee Y.H., Lee M.K., Lee S.H., Kang D.W., Kwon S.J., Oh H.K., Yoon S.Y. (2011). 2011 The National Survey of the Disabled Persons.

[9] National Spinal Cord Injury Statistical Center: Recent Trends in Causes of Spinal Cord Injury. https://www.nscisc.uab.edu/Public_Pages/ReportsStats.

[10] Fann J.R., Bombardier C.H., Richards J.S., Tate D.G., Wilson C.S., Temkin N. (2011). Depression after spinal cord injury: Comorbidities, mental health service use, and adequacy of treatment. Arch. Phys. Med. Rehabil..

[11] Tuner R., McLean R.D. (1989). Physical disability and psychological distress. Rehabil. Psychol..

[12] Rachel L.G., Cynthia L.R., Robert E.M. (2008). Posttraumatic stress disorder and major depression in veterans with spinal cord injury. Rehabil. Psychol..

[13] Hammell K.W. (2010). Spinal cord injury rehabilitation research: Patient priorities, current deficiencies and potential directions. Disabil. Rehabil..

[14] Diana D., Jane M., Linley D. (2013). Applications of telecounselling in spinal cord injury rehabilitation: A systematic review with effect sizes. Clin. Rehabil..

[15] Hammell K. (2004). Exploring quality of life following high spinal cord injury: A review and critique. Spinal Cord.

[16] Duggan C.H., Dijkers M. (2001). Quality of life after spinal cord injury: A qualitative study. Rehabil. Psychol..

[17] Henwood P., Ellis J.A. (2004). Chronic neuropathic pain in spinal cord injury: The patient’s perspective. Pain Res. Manag..

[18] Foreman P.E., Cull J., Kirkby R.J. (1997). Sports participation in individuals with spinal cord injury: Demographic and psychological correlates. Int. J. Rehabil. Res..

[19] Gioia M.C., Cerasa A., Luccente L.D., Brunelli S., Castellano V., Traballesi M. (2006). Psychological impact of sports activity in spinal cord injury patients. Scand. J. Med. Sci. Sports.

[20] Li L., Moore D. (1998). Acceptance of disability and its correlates. J. Soc. Psychol..

[21] Kreuter M., Suulivan M., Dahllöf A.G., Siösteen A. (1998). Partner relationships, functioning, mood and global quality of life in persons with spinal cord injury and traumatic brain injury. Spinal Cord.

[22] Sherrill C. (2004). Adapted Physical Activity, and Sport: Cross-Disciplinary and Lifespan.

[23] Roth K., Zittel L., Pyfer J., Auxter D. (2016). Principle and Methods of Adapted Physical Education and Recreation.

[24] Woody R. (1980). Encyclopedia of Clinical Assessment.

[25] Baik K., Suh Y.T., Kim Y.S. (2014). The relationship among acceptance of disability, self-esteem and happiness of people with physical disability in daily life sports clubs. Korean J. Adapt. Phys. Act..

[26] Duggan C.H., Dijkers M. (1999). Quality of life-peaks and valleys: A qualitative analysis of the narratives of persons with spinal cord injury. Can. J. Rehabil..

[27] Schiaffino K.M., Revenson T.A. (1995). Why me? The persistence of negative appraisals over the course of illness. J. Appl. Soc. Psychol..

[28] Noreau L., Shephard R.J., Simard C. (1993). Relationship of impairment and functional ability to habitual activity and fitness following spinal cord injury. Int. J. Rehabil. Res..

[29] Rauch A., Bickenbach J., Reinhardt J.D., Geyh S., Stuki G. (2010). The utility of the ICF to identify and evaluate problems and needs in participation in spinal cord injury. Top. Spinal Cord Inj. Rehabil..

[30] Heinemann A.W., Shontz F.C. (1982). Acceptance of disability, self-esteem, sex role identity, and reading aptitude in deaf adolescents. Rehabil. Couns. Bull..

[31] Linkowski D.C., Dunn M.A. (1974). Self-concept and acceptance of disability. Rehabil. Couns. Bull..

[32] Oliver M., Hasler F. (1987). Disability and self-help: A case study of the Spinal Injuries Association. Disabil. Handicap. Soc..

[33] Landstad B.J., Hedlund M., Kendall E. (2020). Practicing in a person-centred environment–self-help groups in psycho-social rehabilitation. Disabil. Rehabil..

[34] Lee M.S., Stephen J.L. (2011). Effect of Participation in sport activity on social support and disability acceptance in individuals with spinal cord injury. Korean J. Adapt. Phys. Act..

[35] Kim C.K., Lee H.S. (2003). Effect of rehabilitation sports program on self-efficacy, sport understanding and sport participation in spinal cord injury persons. J. Sport Leis. Stud..

[36] Fliess-Douer O., Vanlandewijck Y.C., Van der Woude L.H. (2013). Reliability and validity of perceived self-efficacy in wheeled mobility scale among elite wheelchair-dependent athletes with a spinal cord injury. Disabil. Rehabil..

[37] Lee M.S. (2010). Effect of sitting badminton participation on self-efficacy and psychological well-being in individuals with spinal cord injury. Korean J. Adapt. Phys. Act..

[38] Adnan Y., McKenzie A., Miyahara M. (2010). Self-efficacy for quad rugby skills and activities of daily Living. Adapt. Phys. Act. Q..

[39] Ablon J. (2002). The nature of stigma and medical conditions. Epilepsy Behav..

[40] Rodakowski J., Skidmore E.R., Rogers J.C., Schulz R. (2012). Does social support impact depression in caregivers of adults ageing with spinal cord injuries?. Clin. Rehabil..

[41] Ogletree B.T., Bull J., Drew R., Brotherson M.J. (2001). Team-based service delivery for students with disabilities: Practice options and guidelines for success. Interv. Sch. Clin..

[42] Ljungberg I., Kroll T., Libin A., Gordon S. (2011). Using peer mentoring for people with spinal cord injury to enhance self-efficacy beliefs and prevent medical complications. J. Nurs..

[43] Cohen S. (2004). Social relationships and health. Am. Psychol..

[44] Villie I., Crost M., Ravaud J.F., Tetrafigap Group (2011). Disability and a sense of community belonging a study among tetraplegic spinal cord injured persons in France. Soc. Sci. Med..

[45] Mael F., Ashforth B. (1992). Alumni and their alma mater: A partial test of the reformulated model of organizational identification. J. Organ. Behav..

[46] Rude S.S., Burham B.L. (1995). Connectedness and neediness: Factor of the DEQ and SAS dependency scales. Cogn. Ther. Res..

[47] Cha J.E. (1996). A Study for the General Self-Efficacy Scale Development. Master’s Thesis.

[48] Bandura A. (1977). Self-efficacy: Toward a unifying theory of behavioral change. Psychol. Rev..

[49] Kim A.Y. (1997). A study on the academic failure–tolerance and its correlates. Korean J. Educ. Psychol..

[50] Seo E.H. (2008). Self-efficacy as a mediator in the relationship between self-oriented perfectionism and academic procrastination. Soc. Behav. Personal..

[51] Linkowski D.C. (1971). A scale to measure acceptance of disability. Rehabil. Couns. Bull..

[52] Linkowski D.C., Groomes D.A.G. (2007). Examining the structure of the revised acceptance of disability scale. J. Rehabil..

[53] Kim H.Y., Jo S.J. (2009). Predicting employment outcomes among industrially injured workers from acceptance of disability, severity, and location of disability. J. Vocat. Rehabil..

[54] Kim W.H. (1994). Organizational identification model: Relations of organizational identification and antecedent variables, consequent variables. Korean J. Ind. Organ. Psychol..

[55] Diener E., Larson R.J., Levine S., Emmons R. (1985). Intensity and frequency: Dimensions underlying positive and negative affect. J. Personal. Soc. Psychol..

[56] Kwon S.M. (2013). Positive Psychology.

[57] Hayes A.F. (2012). PROCESS: A Versatile Computational Tool for Observed Variable, Moderation, and Conditional Process Modeling. http://www.afhayes.com/public/process2012.pdf.

[58] Hu L., Bentler P.M. (1999). Cutoff criteria for fit indexes in covariance structure analysis: Conventional criteria versus new alternatives. Struct. Equ. Model. Multidiscip. J..

[59] Bentler P.M. (1990). Comparative fit indexes in structural models. Psychol. Bull..

[60] Browne M.W., Cudeck R., Bollen K.A., Long J.S. (1993). Alternative ways of assessing model fit. Testing Structural Equation Models.

[61] Fornell C., Larcker D.F. (1981). Evaluating structural equation models with unobservable variables and measurement error. J. Mark. Res..

[62] Hair J.F., Black W.C., Babin B.J., Anderson R.E., Tatham R.L. (2006). Multivariate Data Analysis.

[63] Hong S., Malik M.L., Lee M.K. (2003). Testing configural, metric, scalar, and latent mean invariance across genders in sociotropy and autonomy using a non-Western sample. Educ. Psychol. Meas..

[64] Nicholls E., Lehan T., Plaza S.L.O., Deng X., Romero J.L.P., Pizarro J.A.A., Carlos Arango-Lasprilla J. (2012). Factors influencing acceptance of disability in individuals with spinal cord injury in Neiva, Colombia, South America. Disabil. Rehabil..

[65] Belgrave F.Z., Walker S. (1991). Predictors of employment outcome of black persons with disabilities. Rehabil. Psychol..

[66] Lee P.W.H., Ho E.S.Y., Tsang A.K.T., Cheng Y.H., Cheng J.C.Y., Leung P.C., Lieh-Mak F. (1985). Psychosocial adjustment of victims of occupational hand injuries. Soc. Sci. Med..

[67] Wright B.A. (1983). Physical Disability: A Psychological Approach.

